# Building up Analgesia in Humans via the Endogenous μ-Opioid System by Combining Placebo and Active tDCS: A Preliminary Report

**DOI:** 10.1371/journal.pone.0102350

**Published:** 2014-07-16

**Authors:** Marcos F. DosSantos, Ilkka K. Martikainen, Thiago D. Nascimento, Tiffany M. Love, Misty D. DeBoer, Heidi M. Schambra, Marom Bikson, Jon-Kar Zubieta, Alexandre F. DaSilva

**Affiliations:** 1 Headache & Orofacial Pain Effort (H.O.P.E.), Department of Biologic and Materials Sciences & Michigan Center for Oral Health Research (MCOHR), School of Dentistry, University of Michigan, Ann Arbor, Michigan, United States of America; 2 Translational Neuroimaging Laboratory, Molecular and Behavioral Neuroscience Institute (MBNI), University of Michigan, Ann Arbor, Michigan, United States of America; 3 Departments of Neurology and Rehabilitation & Regenerative Medicine, Columbia University, New York, New York, United States of America; 4 Department of Biomedical Engineering, The City College of New York, New York, New York, United States of America; University Medical Center Goettingen, Germany

## Abstract

Transcranial Direct Current Stimulation (tDCS) is a method of non-invasive brain stimulation that has been frequently used in experimental and clinical pain studies. However, the molecular mechanisms underlying tDCS-mediated pain control, and most important its placebo component, are not completely established. In this pilot study, we investigated *in vivo* the involvement of the endogenous μ-opioid system in the global tDCS-analgesia experience. Nine healthy volunteers went through positron emission tomography (PET) scans with [^11^C]carfentanil, a selective μ-opioid receptor (MOR) radiotracer, to measure the central MOR activity during tDCS *in vivo* (non-displaceable binding potential, BP_ND_) - one of the main analgesic mechanisms in the brain. Placebo and real anodal primary motor cortex (M1/2mA) tDCS were delivered sequentially for 20 minutes each during the PET scan. The initial placebo tDCS phase induced a decrease in MOR BP_ND_ in the periaqueductal gray matter (PAG), precuneus, and thalamus, indicating activation of endogenous μ-opioid neurotransmission, even before the active tDCS. The subsequent real tDCS also induced MOR activation in the PAG and precuneus, which were positively correlated to the changes observed with placebo tDCS. Nonetheless, real tDCS had an additional MOR activation in the left prefrontal cortex. Although significant changes in the MOR BP_ND_ occurred with both placebo and real tDCS, significant analgesic effects, measured by improvements in the heat and cold pain thresholds, were only observed after real tDCS, not the placebo tDCS. This study gives preliminary evidence that the analgesic effects reported with M1-tDCS, can be in part related to the recruitment of the same endogenous MOR mechanisms induced by placebo, and that such effects can be purposely optimized by real tDCS.

## Introduction

Although the neuromechanisms of placebo analgesia are well linked to opioid release in both forebrain structures and descending antinociceptive systems [1]–[4], direct recruitment and optimization of this endogenous resource have been a challenge [5], [6]. Recently, multiple modulatory techniques have been investigated to non-invasively target pain related regions in the brain, and nowadays one of the most frequently used in research is transcranial Direct Current Stimulation (tDCS). Its surge in clinical and scientific reports worldwide is in part due to its low cost and simple operation. tDCS is a method of cortical excitability modulation based on the application of a weak electrical current that flows between electrodes directly applied to the scalp. It has been presented as a relatively effective procedure for neurophysiological experiments, with few adverse events when the safety guidelines are followed [7]. In the most conventional setup for pain research, the anode (positive pole) is placed over the area of the primary motor cortex (M1) and the cathode (negative pole) over the supra-orbital (SO) region [8], [9]. Regarding the analgesic effects, significant results have been reported in various persistent pain syndromes, such as fibromyalgia [10], central pain in traumatic spinal cord injury [11], and chronic migraine [12]. However, neither the analgesic outcomes with tDCS are consistent across studies [13]–[15], nor its endogenous neuromechanisms well understood.

Functional magnetic resonance imaging (fMRI) and positron emission tomography (PET) with blood flow tracers have provided some important information regarding pain modulatory effects of tDCS on cortical and subcortical activity [16]–[18]. Lately, PET has extended our knowledge of molecular mechanisms in the brain in vivo when appropriate radiotracers are utilized. Maarrawi and colleagues, using the “non-selective” opioid receptor radiotracer [^11^C]diprenorphine, showed that surgical motor cortex stimulation (MCS) provided analgesic relief in eight neuropathic pain patients with concurrently reduction in opioid receptor availability in the periaqueductal gray matter (PAG), prefrontal cortex (PFC), anterior mid-cingulate cortex (aMCC), and cerebellum. These data were interpreted as reflecting MCS-induced release of endogenous opioid peptides (Maarrawi, 2007). Consistent with those findings, the analgesic effects produced by non-invasive motor cortex stimulation with repetitive transcranial magnetic stimulation (rTMS) were antagonized by the opioid receptor antagonist naloxone [19]. Interestingly, placebo analgesic effects are also blocked by naloxone [20], [21].

We have recently reported significant acute reductions in μ-opioid receptor (MOR) availability in pain-related regions during a single session of real tDCS in a postherpetic neuralgia patient [22]. The μ-opioid system is the most important mechanism involved in the regulation of nociceptive signals, and specific target of several opioid analgesics currently available for clinical use. The case report utilized [^11^C]carfentanil, a selective MOR radiotracer, and the application of real tDCS was associated with significant changes in thermal pain thresholds. Those preliminary findings suggested that clinical outcomes observed with tDCS could be positively associated with activation of the MOR system, which has been similarly reported in placebo studies [1], [3], [5], [6]. Nevertheless, there remain unknowns regarding the endogenous placebo mechanisms contribution to the final tDCS-induced analgesia.

In this pilot study, we investigated the immediate effects of placebo and its optimization by subsequent real tDCS on MOR mediated neurotransmission and thermal pain thresholds in a group of healthy subjects, applying the M1-SO tDCS montage. We hypothesize that placebo tDCS produces immediate activation of the MOR system, which could be optimized by real tDCS in order to build up an effective analgesic outcome.

## Materials and Methods

### 2.1 Subjects

Nine right-handed healthy volunteers (five males and four females), mean age 44±16, were studied. The exclusion criteria included: A) systemic medical illnesses; B) presence of chronic pain disorders; C) recent surgery or trauma (<6 months); D) use of narcotic analgesics (<6 months); E) major psychiatric illnesses (e.g., schizophrenia, major depression, suicidal ideation, or substance abuse); and F) any PET or MRI contraindications. All volunteers were initially screened to obtain the medical history and to investigate possible contra-indications to MRI, PET or tDCS. After the initial evaluation, they underwent one MRI, and two PET scans. All neuroimaging exams were acquired in different days. The MRI exams were acquired prior to the PET exams. The subjects recruited were not under pharmacological treatment for any condition and no medication was administered prior to the experiments. This research study was conducted in accordance with the bioethical rules for studies involving human beings of the WMA (World Medical Association)––Declaration of Helsinki (1990). All procedures adopted were approved by the University of Michigan Investigational Review Board for Human Subject Use (IRB # 24607) and the Radioactive Drug Research Committee of the US Food and Drug Administration. All subjects gave written informed consent prior to the participation in this study.

### 2.2 Neuroimaging

We used a radiotracer with specific affinity for μ-opioid receptors, [^11^C]carfentanil. Each participant underwent one baseline and one tDCS PET scan using a Siemens (Knoxville, TN) HR+ scanner in 3D mode (reconstructed images have a full-width at half maximum (FWHM) resolution of ∼5.5 mm-in-plane and 5.0 mm axially). Both scans have a total duration of 90 minutes. Synthesis of high specific activity [^11^C]carfentanil was produced by the reaction of [^11^C]methyliodide and a non-methyl precursor [23], [24]. Each [^11^C]carfentanil dose (15±1 mCi, ≤0.05 µg/kg) was administered in a bolus (50% of dose)/continuous infusion protocol to more rapidly achieve steady-state levels. PET images were reconstructed using interactive algorithms into a 128×128 pixel-matrix in a 28.8 cm diameter field of view (FOV). Twenty-eight image frames were obtained and co-registered to one another. They were corrected for motion and decay [25]. Dynamic image data for each scan were converted on a voxel-by-voxel basis into two sets of parametric images using a modified Logan graphical analysis using the occipital cortex as the reference region [26]. First, a tracer transport measure (K_1_ ratio) was used for MRI co-registration and normalization procedures that were applied to the receptor measure. The receptor-related measure, non-displaceable binding potential BP_ND_, or receptor availability *in vivo*, is proportional to B_max_/K_d_ (B_max_ = receptor concentration, K_d_ = receptor-ligand dissociation constant).

A T1-weighted anatomical MRI scan was acquired on a 3 Tesla scanner (General Electric, Milwaukee, WI). The MRI acquisition utilized the following sequence parameters: axial spoiled-gradient recalled (SPGR) 3D acquisition, 15.63 bandwidth, repetition time [TR] = 9.2 ms, echo time [TE] = 1.9 ms, inversion recovery preparation 500 ms, flip angle = 15°, 25/26 FOV, number of excitations [NEX] = 1, 144 contiguous slices, 1.0 mm slice thickness, 256×256 matrix.

Images were anatomically standardized into template space using Statistical Parametric Mapping (SPM8) software by A) co-registering the MR scan and K_1_ scans; B) normalizing the MR scan to the Montreal Neurological Institute (MNI) coordinate system using VBM8 toolbox in SPM8 (http://dbm.neuro.uni-jena.de/vbm8/) and C) applying the resulting deformation matrix to the PET images. Co-registration and normalization accuracy was verified by comparing the transformed MR and PET images to the MNI atlas template.

### 2.3 Transcranial Direct Current Stimulation (tDCS)

The first PET consisted of a baseline scan, when [^11^C]carfentanil was intravenously administered, according the protocol described before, but no other intervention occurred. However, to introduce the subjects to the tDCS protocol during this initial PET, the apparatus was placed on patients' head; and to avoid any suspicious thoughts of deceptive interventions, we showed the end of the electrodes disconnected from the device. During the second PET scan, both placebo and real tDCS were performed. For this purpose, we used a battery-driven constant current stimulator (Soterix Medical 1×1 tDCS) with a pair of conductive-rubber electrodes. Placebo tDCS was applied during the early phase of the PET study (15 to 35 min post-tracer administration), while real tDCS was performed during the late PET phase (60 to 80 min).

**Figure 1:**
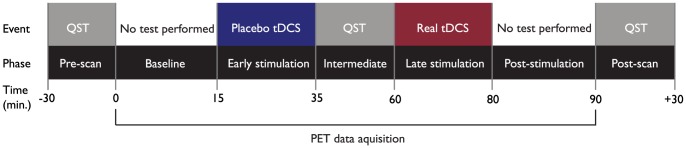
Experimental design used in the second PET scan. Placebo tDCS was applied during the early PET phase (15 to 35 min post-tracer administration) and real tDCS during the late PET phase (60 to 80 min). QST was performed before the PET, in the period between placebo and real tDCS and immediately after the PET.

The experimental design used in this study is illustrated in Figure 1. During placebo stimulation, 2 mA of tDCS was applied for the initial and final 30 seconds of a 20 minutes session, as sensations arising from tDCS are usually observed at the beginning and end of application [27]. The anode was positioned over the superficial area corresponding to the right M1 or C4 position, using the 10/20 system of electrode placement and the cathode was placed over the contralateral supraorbital (SO) region. It is worth mentioning that due to the size of the electrodes, the area contacting the hand representation in the somatosensory cortex (postcentral gyrus) was also likely covered. The same method was used for real tDCS; however, the current was applied for the entire 20 minutes session. Each electrode was enclosed in a 35 cm^2^ sponge soaked with approximately 12 mL of saline solution (6 mL per side) before the PET. We used saline solutions with lower concentrations of NaCl (15 mM) for irrigation. Due to the long time needed for the PET session, we developed a novel method to optimize contact quality of electrodes during the whole period of stimulation to avoid abrupt increases in the overall resistance that could lead to abnormal sensations or burning during both placebo and real tDCS. The system consisted of two syringes, each one connected to the anodal and cathodal sponges by two long cannulas with cross-perforations at their near ends to evenly distribute saline solution through the sponge extension. When contact quality decreased during PET/tDCS session, as demonstrated in the SMARTScan tool of the device (Soterix, NY), our investigators remotely added saline solution to the sponges until reading of contact quality returned to optimal levels. We used 5×7 cm electrodes following the recommendations of previous studies [8], [28]. To assess the safety of the procedure each volunteer was also requested to complete a questionnaire of adverse events at the end of the second PET scan. Following this procedure, subjects were asked if they could distinguish placebo from real tDCS. All volunteers were blind to the treatment conditions.

### 2.4 Quantitative Sensory Testing (QST)

QST was performed on both sides of the face, in order to detect variations in the cold and heat pain thresholds related to both placebo and real tDCS. Considering that this is the first study evaluating the effects of placebo and real tDCS on the μ-opioid system, it is also important to determine if the clinical effects of tDCS are homunculus-specific or occur in both sides of the face. We used Pathway Pain and Sensory Evaluation System (Medoc, Israel) to apply thermal stimuli and record subject's response. For this purpose, we used a 16×16 mm thermode, which contains an extended cable length. This procedure was carried out in three distinct time points during the second PET scan: 1- before the exam started, 2- between placebo and real tDCS (35 to 60 min from radiotracer administration) and 3- after real tDCS. Heat and cold stimuli were applied to the third division of the trigeminal nerve (V3), bilaterally. Pain thresholds were assessed with the “method of limits”. In this method, the intensity of a stimulus is gradually increased until the subject perceives a predefined sensation (e.g. pain) and manually stops the stimulation [29]–[32]. Following a warning signal, the temperature increased from an adaptation temperature of 30°C with a ramp rate of 1°C/s. The volunteer was instructed to press a button as fast as possible at the moment the stimulus became painful. Three assessments were obtained for each stimulus, with an interstimulus interval of 40 seconds and heat and cold pain thresholds were calculated by taking the average temperature of the three assessments. All participants were instructed to report any unexpected sensation arising from QST during the experiment.

### 2.5 Data analysis

MOR activation was measured as the reduction in MOR BP_ND_ from baseline to the given experimental condition (placebo or real tDCS). The effects of placebo and real tDCS were examined separately using paired t tests. Due to the explorative nature of this study, threshold for significance was set at p≤0.001, >40 voxels, for *a priori* hypothesis regions (areas shown to be involved in MOR-mediated pain control in previous studies). The significant clusters were extracted and the average cluster values were used to test the potential associations between the MOR BP_ND_ data and selected variables. Pearson's coefficient correlation was applied to determine the degree of association between placebo and real tDCS MOR activation in each significant cluster found.

Due to the small sample size, wide and non-Gaussian distribution of the data, non-parametric statistical tests were chosen to evaluate the tDCS effects on the heat and cold pain thresholds. Although it is an explorative, pilot study, a more conservative statistical methodology was applied, using the Friedman ANOVA and Nemenyi multiple comparisons test, which permits to adequately control the type I error at the level of 5%. Friedman ANOVA was applied to evaluate the presence of significant variations in the cold and heat pain thresholds related to placebo and real tDCS. When the null hypothesis could be rejected at p<0.05, the Nemenyi test for multiple comparisons was used to verify whether significant differences existed between any pair of observations (baseline×sham tDCS×real tDCS). We set the significance level at 5% and used the software SAS 6.11 (SAS Institute, Inc., Cary, NC) to conduct this part of the statistical analysis.

## Results

All participants successfully completed the study. No major adverse events related to PET, tDCS or QST were reported. None of the subjects could accurately differentiate placebo and real tDCS at the end of the PET session.

### 3.1 Placebo and Real tDCS Effects on Central μ-Opioid Activation

**Figure 2:**
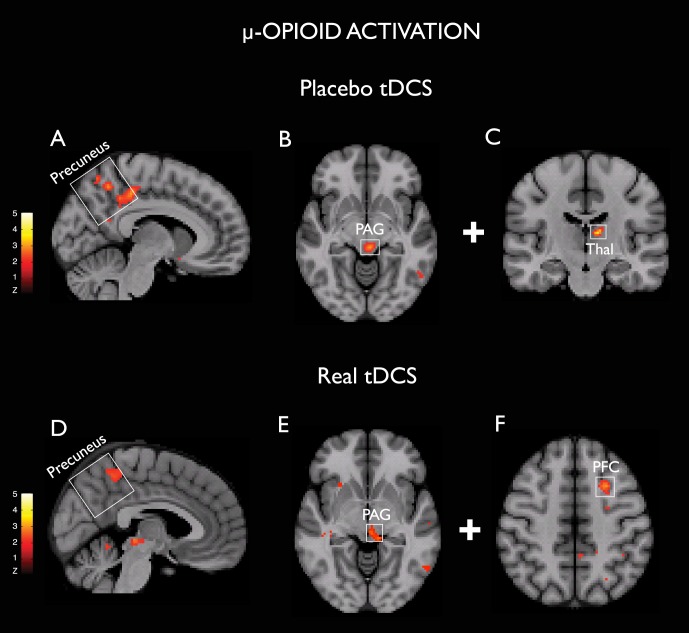
Changes in the μ-opioid receptor availability induced by placebo (A–C) and real (D–E) tDCS. A and D, Representation of precuneus MOR activation in the sagittal plane. B and E, PAG MOR activation in the axial plane. C, Left thalamus (Thal) MOR activation in the coronal plane. F, Left prefrontal cortex (PFC) MOR activation in the axial plane. All images are radiological in orientation, threshold T 3–8.

A significant placebo tDCS-induced MOR activation was observed in the right precuneus [(6, −34, 46), 1040 mm^3^, Z = 3.5], PAG [(−4, −30, −6, 712) mm^3^, Z = 3.9] and left thalamus [(−10, −18, 12), 280 mm^3^, Z = 4.2] (Figure 2 A–C). No significant clusters were detected in the opposite contrast (i.e., MOR deactivation during placebo tDCS). During subsequent real tDCS phase, clusters showing MOR activation were also found in the left precuneus [(−4, −46, 52), 952 mm^3^, Z = 3.1], PAG [(−2, −26, −4), 472 mm^3^, Z = 3.4], and left PFC [(−26, 12, 48), 744 mm^3^, Z = 3.5] (Figure 2 D–F).

**Figure 3:**
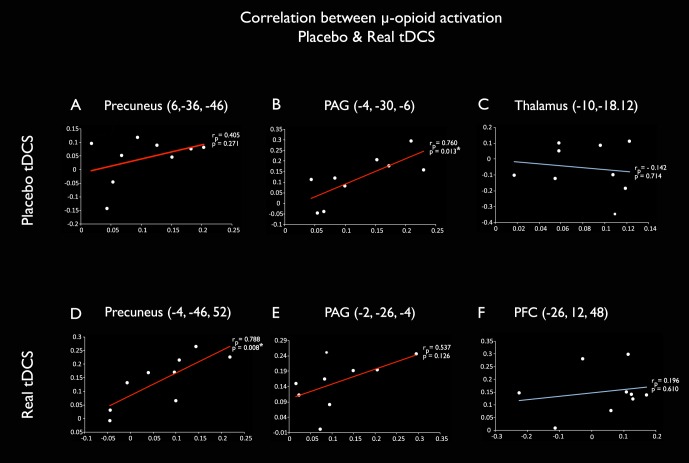
Correlation between placebo and real tDCS-induced MOR activation. MOR BP_ND_ during placebo (x axis) and real (y axis) tDCS for each subject in the clusters of μ-opioid activation induced by placebo (A–C) and real (D–F) tDCS. The same clusters are illustrated in the figure 2. Positive correlations can be observed in precuneus and PAG (red lines) but not in thalamus and PFC (blue lines). Statistically significant values at p<0.05 were found in the PAG cluster activated during placebo tDCS (r_p_ = 0.760, p = 0.013, 3B) and in the precuneus cluster activated during active tDCS (r_p_ = 0.788, p = 0.008, 3D).

Placebo and real MOR activations showed positive correlation in the PAG and precuneus (Figure 3 A, B, D and E), but not in the thalamus or PFC (Figure 3 C and F). Significant correlations were observed in the following coordinates: PAG [(−4, −30, −6) r_p_ = 0.760, p = 0.013] and left precuneus [(−4, −46, 52), r_p_ = 0.788, p = 0.008] clusters (Figure 3 B and D, respectively),

### 3.2 Analgesic tDCS Effects on Heat and Cold Pain Thresholds

**Figure 4:**
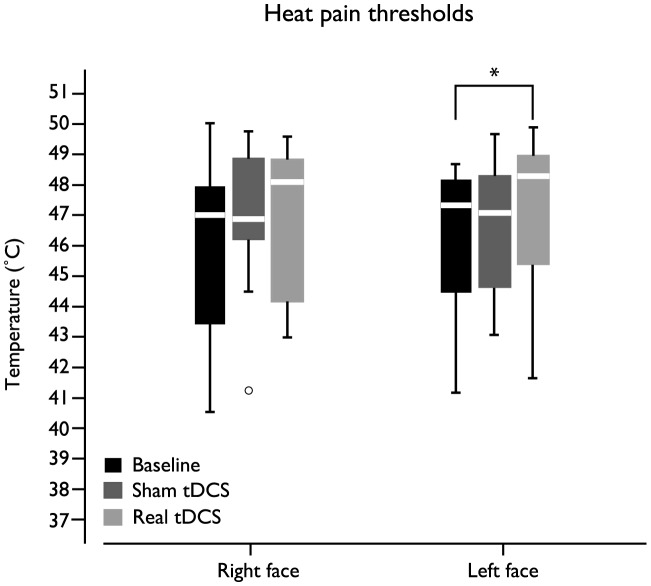
Box plot representing tDCS (placebo and real) effects in the heat pain thresholds of both sides of the face. Statistically significant changes occurred in the heat pain thresholds of the left face (*p* = 0.032).

**Figure 5:**
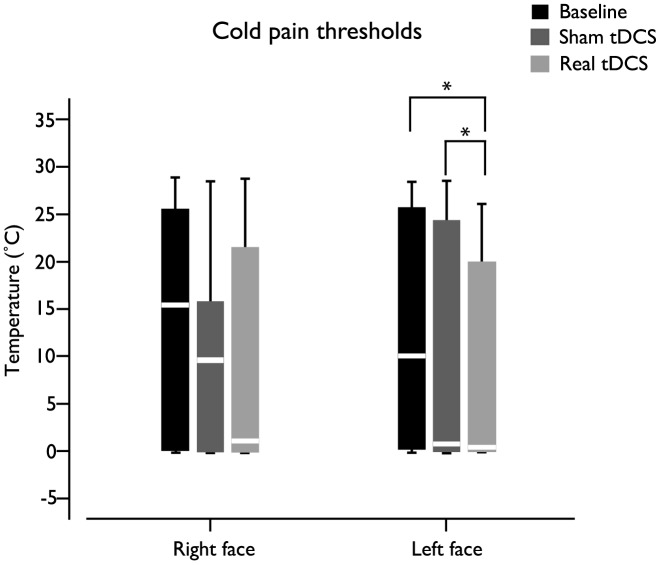
Box plot illustrating cold pain thresholds variations related to placebo and real tDCS. Statistically significant changes occurred in the cold pain thresholds of the left face (*p* = 0.012) throughout the experiment.

Overall, the effects of real tDCS were more pronounced than placebo tDCS effects, for both heat and cold pain thresholds (Figures 4 and 5, respectively).

According to Friedman ANOVA, statistically significant changes occurred in the left face heat (*p* = 0.032) and cold (*p* = 0.012) pain thresholds throughout the experiment. Nemenyi test for multiple comparisons identified that the left face heat pain threshold was significant higher after real tDCS, when compared to baseline (Figure 4). The same test showed that the left face cold pain threshold was significantly higher; implying increased cold pain tolerance, after real tDCS, when compared to placebo tDCS and baseline.

## Discussion

We tested the hypothesis that placebo tDCS induces immediate effects on endogenous μ-opioid receptor (MOR)-mediated neurotransmission, and that such analgesic effects can be optimized at molecular and clinical levels when real tDCS is delivered. Our results indicate that placebo and real tDCS induced similar and positively correlated MORs activations in the PAG and precuneus, as well as dissimilar activations in the thalamus and PFC respectively (Figures 2 and 3); which led to build up of effective thermal pain analgesia when real tDCS was subsequently added to the placebo experience.

The contribution of the μ-opioid system in brain stimulation analgesia has been suggested with MCS [33], TMS [19], [34] and more recently with tDCS [22]. However, the association of the μ-opioid transmission induced by real tDCS neuromodulation with the placebo experience and the analgesic outcomes have not been explored. The involvement of PAG and precuneus in central pain processes has been shown in functional neuroimaging studies in acute and chronic pain, including migraine [35]–[42]. PAG is a region with high levels of μ-opioid receptors and one the most important areas for opioid-mediated anti-nociception [2], [35], [36], [43]. This particular midbrain region is considered central in placebo mechanisms, given its involvement in descending pain analgesia during placebo administration [1], [4]. Precuneus activation during placebo anticipation has been reported in the absence of pain, and deactivation during placebo administration [44], potentially reflecting the participation of this region in the integration of cognitive and sensory assessments related to pain signal. Of note, strong precuneus activation was reported after intravenous administration of the MOR agonist remifentanil during fMRI, indicating that this region may also be important in opioidergic pain modulation [45]. Furthermore, the increased connectivity between precuneus and PAG recently demonstrated after active electroacupunture, suggests the participation of both regions in different modalities of pain modulation [46]. More recently, PET studies have confirmed those findings by demonstrating the activation of MOR neurotransmission during placebo administration in the PAG and thalamus, among other brain regions [1], [4]. Here, the PAG, precuneus, and thalamus activations observed for the period of our early PET/tDCS protocol are in agreement with the notion that placebo brain stimulation prompts endogenous MOR neurotransmission in areas implicated in pain regulation.

Remarkably, when we subsequently added the real tDCS, there were similar MOR activations in the precuneus and PAG (Figure 2D and 2E), which were positively correlated with the prior placebo tDCS (Figure 3D and 3E). One interpretation is that the more placebo induced μ-opioid neurotransmission in the PAG and precuneus, the stronger their regional activation during real tDCS. This observation suggests that successful M1-tDCS analgesia depends in part on individual susceptibility to mobilize μ-opioid activity during placebo. An alternative interpretation, given we are assessing correlation and not causality, is that the two responses are increased but unrelated phenomena.

Conversely, this finding does not signify that MOR neurotransmission elicited by real tDCS is solely based on the patient's belief of the therapeutic effect. In fact, the supplementary MOR activations noted in the thalamus and PFC, respectively following placebo and real tDCS, did not show any statistical correlation or tendency (Figure 3 C and F).

The MOR activation in the PFC is associated with higher order cognitive functions such as attention, decision-making and working memory [47], [48]. Different subregions of the PFC have been implicated in pain perception and/or modulation with a critical role in the emotional processing of pain [49]–[56]. Increased cerebral perfusion has been recently demonstrated during anodal tDCS applied to the dorsolateral PFC [57]. Moreover, it has been shown that PFC stimulation produces its analgesic effects via endogenous opioid release, as it is reversed by naloxone [34], [58]. Finally, MCS induces opioid activation in the PFC and PAG also correlates with pain improvement [33]. Interestingly, using a high-resolution tDCS computational model, our group has previously demonstrated peaks of current flow in the bilateral PFC, suggesting that M1-tDCS can potentially directly target the neuronal activity of this area [12]. Hence, we speculate that indirect and direct PFC modulation by M1-tDCS might contribute to the significant regional endogenous μ-opioid release, which in turn, would contribute to the higher analgesic effects of real tDCS.

Overall, we found significant improvement of cold and heat pain tolerance following real tDCS, consistent with other studies [59]–[65]. The large variability in the QST values found in our study, especially for cold pain thresholds, are similar to other reported values [66], [67]. Interestingly, significant changes in the thermal pain thresholds were only observed in the face contralateral to the side where tDCS was delivered. These findings could be explained by a direct effect of conventional M1-tDCS in cortical and even subcortical brain structures ipsilateral to the stimulation. Nonetheless, due to the large dimensions of the electrodes other areas covering the cortical homunculus could also have been stimulated [12]. High-definition tDCS may provide in the future additional information about the sole contribution of motor and somatosensory stimulation to placebo and real MOR mechanisms [68]. In the current research protocol, tDCS was delivered on the non-dominant motor cortex. It is possible that stimulation of the dominant cortex could have produced different neuroimaging and clinical outcomes. In addition, the effects placebo and active tDCS in other dermatomes, outside the trigeminal territory remains to be explored.

It is important to emphasize that our protocol aimed to investigate the endogenous effect of placebo tDCS and its immediate contribution and optimization by subsequent real M1-SO tDCS, not the opposite. Although randomization and other montages and currents would also provide important information regarding other neuromechanisms of tDCS [27], [69], these aims were outside the scope of our study. Furthermore, it is possible to hypothesize that increasing the sample size might expand our results to a broader set of brain structures. Nevertheless, the important finding of this preliminary report is that both placebo and active tDCS induce MOR activation and, consequently, they jointly contribute to the benefits of the treatment in clinical samples.

## Conclusions

In this preliminary report, we demonstrate, in a cohort of healthy subjects, that placebo tDCS produces acute changes in the endogenous MOR-mediated neurotransmission, indicating activation of the analgesic μ-opioid mechanism, and that such effect is optimized at molecular and clinical levels by real tDCS. This suggests that M1-SO tDCS might in part recruit and effectively potentiate the same analgesic resources elicited during placebo experience. Further studies, assessing different study protocols (e.g. naloxone modulation), in larger sample sizes and including chronic pain patients, will be necessary to confirm our findings, and to scrutinize the long-term effects of tDCS on analgesic μ-opioid activation and other brain mechanisms, before its potential therapeutic application in chronic pain relief.
